# JAG1, Regulated by microRNA-424-3p, Involved in Tumorigenesis and Epithelial–Mesenchymal Transition of High Proliferative Potential-Pituitary Adenomas

**DOI:** 10.3389/fonc.2020.567021

**Published:** 2020-12-23

**Authors:** Yiyuan Chen, Bin Li, Jie Feng, Qiuyue Fang, Jianhua Cheng, Weiyan Xie, Chuzhong Li, Sen Cheng, Yazhuo Zhang, Hua Gao

**Affiliations:** ^1^ Beijing Neurosurgical Institute, Capital Medical University, Beijing, China; ^2^ Department of Neurosurgery, Beijing Tiantan Hospital, Capital Medical University, Beijing, China; ^3^ Beijing Key Laboratory of Central Nervous System Injury, Capital Medical University, Beijing, China

**Keywords:** pituitary adenoma, Notch signaling, epithelial–mesenchymal transition, five-tiered prognostic classification of PitNETs, Jagged1 canonical Notch Ligand, miR-424-3p

## Abstract

Pituitary adenomas (PAs) are a neoplastic proliferation of anterior pituitary. Signature of Notch pathway relies upon the histopathological type of PAs. The details of Notch pathway that are involved in the migration and invasion of Pas are still unclear. This paper filters and testifies the relation between Notch signaling pathway and the migration/invasion in subtypes of PAs. The diversity of genes and pathways is investigated based on transcriptome data of 60 patients by KEGG pathway analysis and GSEA. A series of functional experiments demonstrate the role of candidate genes by overexpression and antibody blocking in GH3 cell line. Volcano map and GSEA results exhibit the differential and the priority of Jagged1 canonical Notch Ligand (JAG1) in the Notch pathway combined with clinical features. JAG1 is involved in epithelial–mesenchymal transition (EMT) in PAs by correlation analysis of RNA-seq data. Progression-free survival (PFS) of patients with high JAG1 was shorter than patients with low JAG1 according to follow-up data (P = 0.006). Furthermore, overexpression and antibody blocking experiments in GH3 cell line indicate that JAG1 could promote cell proliferation, migration, and G1/S transition. Double luciferase reporter assay gives manifests that JAG1 is the target gene of miR-424-3p, and mimics or inhibitor of miR-424-3p can regulate the level of JAG1 which, in turn, affects cell proliferation and the levels of MMP2 and VIM in GH3 cell line, respectively. Our study delves into the relation between the Notch signaling pathway and cell proliferation and EMT in PAs, providing a potential treatment through targeting JAG1.

## Introduction

Pituitary adenoma is a neoplastic proliferation of anterior pituitary and accounts for 10–15% of intracranial tumors (1). Except for lactotroph adenomas, the initial therapy for patients with PAs is generally transsphenoidal surgery, while medical therapy is being reserved for those with unfavorable surgical outcome (2). About 35% PAs invade the sphenoid bone or cavernous sinus which are the major factors influencing resectability. The remission rate is only 40–66% in macroadenomas while more than 85% in microadenomas (3). The diagnosis of invasion is mainly based on adenomas extent and invasion of adjacent anatomic structures, especially Knosp staging. Moreover, the inter-rater reliability of grade III/IV tumors is strong, while that of grade I/II is weak (4). It is important to identify the invasive Pas’ growth from lateral growth without invasion because the former is linked to a higher risk of recurrence. In 2013, Trouillas et al. depicted the Five-Tiered prognostic classification of PitNETs including tumor diameter, tumor type, and grading that was based on invasion and proliferation (5, 6). At present, most commonly utilized molecular markers of proliferation categories (PitNETs: 1/2a, 1/2b) are: 1) Ki-67 index: >3%; 2) mitoses count: n >2/10 high power field (HPF), 3) p53 detection positive (10 strongly positive nuclei/10HPF) (7). In addition, pituitary tumor-transforming gene 1 (PTTG1) and c-Myc also play important roles in early pituitary tumorigenesis (8). However, the mechanism of different proliferation categories is still unclear.

Notch signaling pathway has been evolutionarily conserved in mammals, including Notch1–4 receptors and their ligands Delta like canonical Notch ligand (DLL) 1/3/4 and JAG1/2 in Homo sapiens. Aberrant activation of Notch receptor and its ligands have been shown to be linked in cell fate and differentiation, repair-regeneration, tumorigenesis, and metastasis (9). Also, DLLs and JAG are crucial in restricting cell fate, differentiation, and size of neuroendocrine associated secretory cells (10). Studies showed that Notch signaling pathway is associated with the maintenance of stem/progenitor cells in the anterior pituitary gland (11). Specific Notch system components and involvement depend on the histopathological type of PAs, with higher activation in corticotroph adenomas (12). Notch2 and the ligand JAG1 are localized within E-cadherin-positive cells in the marginal cell layer and the main part of the anterior lobe (13). A positive correlation has been reported between expression of Notch2/DLL3 pathway and invasion in somatotroph adenomas, and knockdown-Notch2 noticeably inhibited the cell migration in both GH3 cell line and primary tumor cell (14). Up-regulated Notch3 and JAG1 have been observed in human non-functional PAs, but not in human pituitary glands or hormone-secreting adenomas. In the majority of examined PA samples, an increase has been found in the expression of relative Notch3 and JAG1 mRNA, which may be a crucial factor in the initiation and proliferation of human non-functional adenomas (15, 16).

Increasing evidence has been pointing to alterations in gene expression as a major contributor to identify disease-specific patterns for infrequent recurrent somatic mutations in PAs (17, 18). In this study, 60 patients were divided into two groups in accordance with the Five-Tiered prognostic classification of PitNETs for transcriptome experiments. Through the diversity analysis of total productive maintenance (TPM) values, specific alterations of pathways were filtered. We analyzed the expression profile of Notch signaling pathway combined with pathological and clinical annotations of patients. Furthermore, this paper examines the priority of JAG1 in Notch signaling pathway and verifies its functions that provide potential molecular therapeutics for future therapy.

## Materials and Methods

### Patient Samples and Cell Lines

All samples were obtained following transsphenoidal surgery performed at Beijing Tiantan Hospital from May 2012 to December 2014. Fresh tumor samples were stored in liquid nitrogen. 20 somatotroph/lactotroph adenomas, 21 gonadotroph adenomas and 19 corticotroph adenomas from the study population (age range, 20–69 years) were diagnosed according to the 2017 World Health Organization classification of tumors of endocrine organs. Six normal pituitary glands were obtained from a donation program. The study protocols were approved by the Internal Review Board of Beijing Tiantan Hospital, which was affiliated to Capital Medical University and conformed to the ethical guidelines of the Declaration of Helsinki (no. KY-2013-02).

GH3 cell line was purchased from ATCC and cultured in a humidified incubator at 37°C and 5% CO_2_ in F-12K medium (ATCC, Manassas, VA, USA) supplemented with 2.5% fetal bovine serum and 10% horse serum. JAG1 plasmid and vector were purchased from Origene (RR203938, MD, USA).

### RNA Extractions, Sequencing, RNA-Seq Data Processing, and Analysis

For RNA extractions, patient samples were performed with AllPrep DNA/RNA Mini kit (Qiagen, UK) according to the manufacturer’s instructions. The quantity and quality of RNA were evaluated by RNA Nano6000 assay kit (Aligent Technologies, CA, USA) (RIN >6.8). 3 μg RNA/sample was used for RNA preparations; then the ribosomal RNA by Epicentre Ribo-zeroTM rRNA Removal Kit (Epicentre, USA) was removed. Sequencing library was generated by NEBNext^®^ UltraTM Directional RNA Library Prep Kit (NEB, USA). The library fragments (150–200 bp) were purified by AMPure XP system (Beckman Coulter, Beverly, USA), then assessed by Agilent Bioanalyzer 2100 system. The libraries were sequenced on an Illumina Hiseq X platform, then 150 bp paired-end reads were generated. Reads containing adapters, containing ploy-N and low-quality reads were removed. Clean reads were mapped to the human reference genome (NCBI37/hg19) using hisat2 (v2.0.5) to get read counts/FPKM/TPM for each noticed gene. R package limma (https://git.bioconductor.org/packages/limma) was used to analyze the quantitative differentiation between two identified groups. KEGG pathway enrichment and GO term results were exported from KOBAS (http://kobas.cbi.pku.edu.cn/kobas3/genelist/) website with filtered differential gene data input. R package clusterProfiler (https://guangchuangyu.github.io/software/clusterProfiler) was used to process the GSEA analysis.

### Tissue Microarray Construction and Immunochemistry Staining

A total of 66 formalin-fixed paraffin-embedded tissue blocks were sectioned. Three core biopsies (2.0 mm in diameter) were selected from the paraffin-embedded tissue. The cores were transferred to tissue microarrays using a semi-automated system (Aphelys MiniCore, Mitogen, UK). The microarrays were cut into 4-μm sections and incubated with anti-JAG1 (Mouse monoclonal, 1:500, ab89663, Abcam), anti-MMP2 (Rabbit polyclonal, 1:300, ab97779, Abcam) and anti-Ki-67 (Rabbit monoclonal, 1:400, ab16667, Abcam) primary antibodies. H-score was obtained by multiplying the staining intensity by a constant to adjust the mean to the strongest intensity [H-score = 3 × (percentage of strong staining)] (1.0%, weak; 2.0%, moderate; 3.0%, strong) to give a score ranging from 0 to 300.

### Cell Proliferation, Apoptosis, Cell Cycle, and Migration Assays

GH3 cells were adjusted to a density of 1 × 10^5^ cells/ml. A total of 100 μl of the cell suspension was plated into each well of a 96-well plate and cultured overnight. After transient infection with 3 μg vector or JAG1 plasmid on 1 × 10^6^ cells using Lipo3000 for 24, 48, and 72 h, 20 μl of 3-(4,5-diethylthiazol-2-yl)-5-(3-carboxymethoxyphenyl)-2-(4-sulfophenyl)-2H -tetrazolium, inner salt (MTS) solution was added to each well, and the cultures were further incubated for 4 h. Absorbance was measured at 490 nm using an ELISA plate reader (Thermo, USA). Apoptosis and cell cycle were determined by flow cytometry using Annexin V+PI Detection Kit (BD Pharmingen, CA, USA).

Cell migration was measured using fibronectin and Matrigel-coated polycarbonate filters, respectively, and modified transwell chambers (Corning, MA, USA). GH3 cells (5 × 10^4^ cells) were added into the upper chambers. Migrating cells that adhered to the lower membrane were fixed in 4% paraformaldehyde and stained using hematoxylin (Zhongshan Company, Beijing, China). Experiments were performed in triplicate.

### Reverse Transcription and Quantitative PCR

Microarray hybridization and RT-qPCR were performed as previously described. Total RNA of 30 samples was extracted and purified using the Rneasy^®^Mini Kit (QiaGen, Hilden, Germany) following the manufacturer’s instructions. RT-qPCR was performed on a QuantStudio5 (Applied biosystems, Singapore). The fold-change in differential expression for each gene was calculated using the comparative CT method (2^−ΔΔCT^ method) in R package with “PCR” functions (https://github.com/MahShaaban/pcr), a GAPDH reference gene, and the “1/2a” stage reference group (19). The primers of genes were listed in **Table S1**.

### SDS-PAGE and Western Blot Analyses

Samples (up to 10 mg) were lysed in lysis buffer containing 1% Nonidet P-40 (Calbiochem, Merk, Darmstadt, Germany) and protease and phosphatase inhibitor cocktails (Roche, IL, USA) overnight at 4°C. Total extracts were centrifuged at 12,000 g for 30 min at 4°C, and protein concentration was determined with the BCA method (Pierce Biotechnology, IL, USA). A total of 40 μg of protein per lane was loaded onto 10% Bis-Tris SDS-PAGE gels, separated electrophoretically, and blotted onto polyvinylidene fluoride membranes (Merk). The blots were incubated with antibodies against anti-JAG1 (1:2,000), anti-MMP2 antibody (1:1,500), anti-VIM (Rabbit monoclonal, ab92547, 1:1,000, Abcam), and anti-SNAI1 (Rabbit polyclonal, 1:2,000, ab180714, Abcam) followed by a secondary antibody (1:8,000) tagged with horseradish peroxidase (Santa Cruz Biotechnology). Blots were visualized by enhanced chemiluminescence, and densitometry was performed using a fluorescence image analyzer (Amersham Imager 600, GE, MA, USA). GAPDH was used as a loading control.

### Dual-Luciferase Reporter Gene Assay

The miRTarBase (http://mirtarbase.mbc.nctu.edu.tw/php/index.php) was used to predict the target mRNAs of miRNAs. JAG1 sequences were acquired from Ensembl (http://asia.ensembl.org/index.html). Interacting miRNA and mRNA sequences were analyzed by Global Align in Blast (https://blast.ncbi.nlm.nih.gov). JAG1 sequences harboring either the wild-type (WT) or mutated binding sites (MT) for miR-424-3p were amplified by PCR and cloned into the pmirGLO luciferase vector (Promega, Madison, WI, USA) to create wild-type or mutant pmirGLO-JAG1 vectors, respectively, using the Lipofectamine 3000 transfection kit (Promega, Madison, WI, USA). GH3 cells harboring the wild-type or mutant pmirGLO-JAG1 were co-transfected with the mimics or miR-NC, respectively, for 48 h. After transfection, luciferase reporter gene activity was determined following the instructions of the dual-luciferase detection kit (Promega, Madison, WI, USA).

### Statistical Analysis

All statistical analyses were conducted using SPSS Statistics Version22 (IBM Corporation, Armonk, New York, USA). An unpaired Student’s test and a chi-square (Fisher’s exact) test were used to compare quantitative and qualitative data. *P*-value of less than 0.05 was considered significant.

## Results

### Clinical and Pathological Features in 60 PAs

60 cohort samples for transcriptome were included, with 32 males and 28 females with average age 46.8 ± 1.56 (20–69 years), comprised of five microadenomas (diameter ≤10 mm) and 37 macroadenomas (10 mm<diameter ≤ 40 mm) and 22 giant (diameter >40 mm) in **Table 1**. According to Five-Tiered prognostic classification of PitNETs, patients were identified into two phenotypes: 30 non-proliferative (1/2a) and 30 proliferative (1/2b); 32 non-invasive (1a/b) and 28 invasive (2a/b). Transsphenoidal surgery included initial surgery (55/60, 91.7%), and five recurrence adenomas cases for surgery (5/60, 8.3%). Recurrence rate was 17/60 (28.3%) according the follow-up data (average 40.5 ± 2.43 months, range 3–71 months). This series included 20 somatotrophs and lactotrophs (PIT1 lineage), 21 gonadotrophs (SF1 lineage) and 19 corticotrophs (TBX19/TPIT lineage).

**Table 1:** The clinical data in 60 patients.

Variables	Invasion		*P* value	Proliferation		*P* value
	Grade 1a/b	Grade 2a/b		Grade 1/2a	Grade1/2b	
Gender			0.628			1
Male	18	14		16	16	
Female	14	14		14	14	
Age (Median, y)			0.121			0.302
<46.8	12	18		13	18	
≥46.8	18	12		17	12	
Tumor diameter			0.001			0.001
Microadenoma	5	0		5	0	
Macroadenoma	27	10		22	15	
Giant	0	18		3	15	
Lineage			0.486			0.697
PIT1(+)	12	8		9	11	
SF1(+)	9	12		10	11	
TPIT(+)	11	8		11	8	
Follow-up Recurrence			0.032			0.045
Yes	5	12		5	12	
No	27	16		25	18	

### Transcriptome in 60 PAs

**Figure 1:**
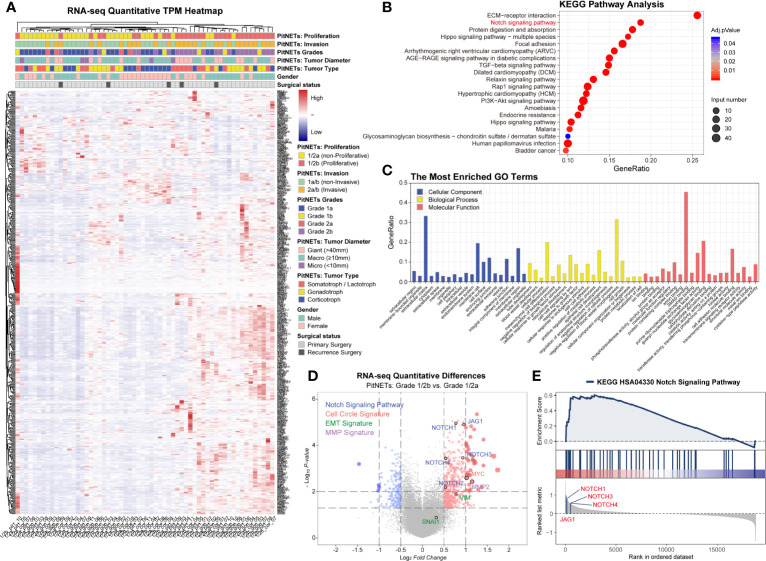
Transcriptome of 60 PA patients. **(A)** Heatmap of unsupervised hierarchical clustering of the top 490 most variable genes. Pathological and clinical annotations were provided. **(B)** KEGG pathway analysis based on the diversity of total productive maintenance (TPM) values (Log_2_FC <−1 or >1, *P* < 0.05). **(C)** The most Enriched GO Terms. **(D)** Volcano map of transcriptome. Notch signaling pathway, cell circle signature, EMT signature and MMP family were provided. *Log_2_FC*<−1 or >1, *P* < 0.05 **(E)** GSEA showed JAG1’s position in Notch signaling pathway.

In this study, we divided 60 patients into two groups according to the Five-Tiered prognostic classification of PitNETs for transcriptome experiments (1/2a *vs.* 1/2b). An unsupervised hierarchical clustering of the top 490 most variable genes revealed two distinct gene-expression profiles corresponding to the discretion of proliferation categories in **Figure 1A**. The heatmap of each pathological type was shown in **Figure S2**. We noticed the inconsistent of high proliferation and invasive patients mainly focused macroadenomas (7/37, 18.9%) and giant adenomas (3/18, 16.7%). There was no correlation between the proliferation categories and gender/pathological type/age.

Through the diversity of total productive maintenance (TPM) values (Log_2_FC>1, P < 0.05), the top three pathways were ECM–receptor interaction, Notch signaling pathway, and protein digestion and absorption by KEGG pathway analysis in **Figure 1B**. The biological process of enriched GO terms focused on the regulation of blood vessel morphogenesis, mesenchymal cell differentiation, epithelial cell development, *etc.* in **Figure 1C**. Volcano map and GSEA both exhibited the priority of JAG1 in Notch signaling pathway in **Figures 1D, E** and **Figure S1** and assured the differential expression of Notch system components involved in the proliferation categories in **Figure S3**: Notch3 in corticotroph and gonadotroph, Notch2 in PIT1 lineage.

### Correlation Analysis of Notch Pathway and Clinical Characterization in 60 Patients

**Figure 2:**
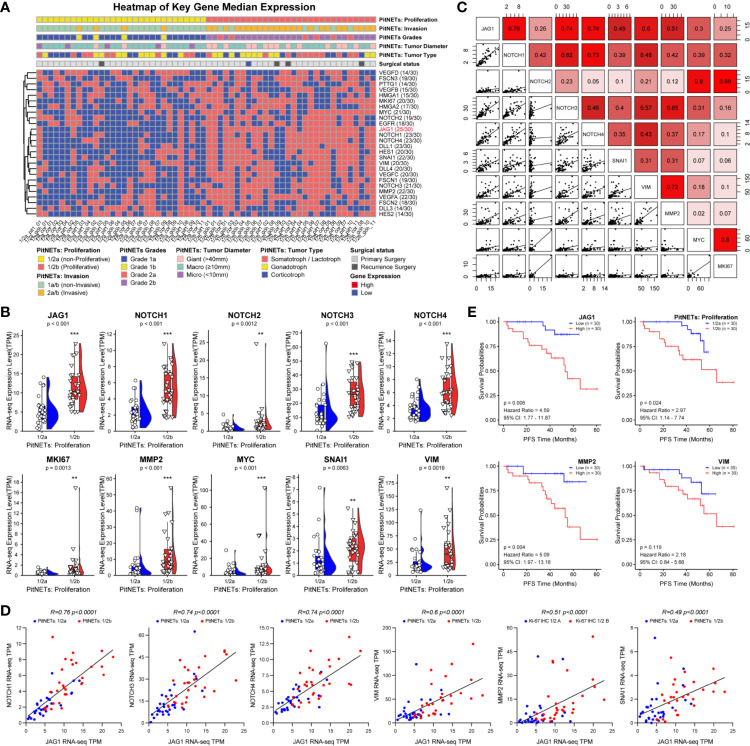
Notch signaling pathway involved into the tumorigenic and recurrence of PA. **(A)** Heatmap of key gene median expression including angiogenesis, high mobility group family and Notch signaling pathway. Pathological and clinical annotations were provided. **(B)** RNA-seq quantitative levels (TPM) of Notch pathway and genes related to EMT. **(C)** The correlation analysis between Notch pathway and EMT. **(D)** The correlation of between JAG1 and Notch receptors, genes related to EMT. *Log_2_FC*<−1 or >1, *P*<0.01 **(E)** PFS time according the levels of JAG1, MMP2, SNAI1 and VIM. *Log_2_FC*<−1 or >1, *P <*0.01. **p < 0.01, ***p < 0.001.

Based on the proliferation categories (1/2a *vs*. 1/2b), we investigated the features of Notch signaling pathway, angiogenesis, high mobility group, migration and invasion in **Figure 2A**. We noticed that JAG1 was the most significant molecule in Notch signaling pathway, followed by Notch1, Notch4, and DLL1 in **Figure S4**. In addition, MMP2, SNAI1, and VEGFA contributed to proliferation and invasion of adenomas. We further filtered and shortened the candidate genes involved in the EMT of PA patients in **Figures 2B, C** and **Figure S5**. As the downstream ligand, the TPM value of JAG1 was positive correlated to SNAI1 (*r* = 0.49, *P* < 0.001), VIM (*r* = 0.6, *P* < 0.001), MMP2 (*r* = 0.51, *P* < 0.001) in **Figure 2C**. Notch2 could promote tumor proliferation through regulating the levels of MYC (*r* = 0.8, *P* < 0.001) and MKI67 (*r* = 0.89, *P* < 0.001). The correlation analysis demonstrated the upstream Notch receptors and downstream target genes might be MMP2, SNAI1, and VIM of JAG1 in **Figure 2D**. Follow-up data showed that high JAG1 (Hazard ratio = 4.59, 95%CI: 1.77–11.87, *P* = 0.006), group 1/2b (Hazard ratio = 2.97, 95%CI: 1.14–7.74, *P* = 0.024) and high MMP2 (Hazard ratio = 5.09, 95%CI: 1.97–13.18, *P* = 0.004) shortened the PFS time in **Figure 2E**. And there was no statistical difference of PFS time according to the level of VIM (Hazard ratio = 2.18, 95%CI: 0.84–5.66, *P* = 0.119). Combined the clinico-pathological characteristics of 60 patients, patients with high JAG1 had larger tumor diameter, more invasive/proliferative potency (PitNETs: 2 & PitNETs: b) than low JAG1 tumor in **Table 2**.

**Table 2:** Association between JAG1 expression and clinico-pathological characteristics.

Variables	JAG1 level		Univariate analysis	
	High^#^ (n = 30)	Low (n = 30)	χ²	P-value
**Age** (years)			1.07	0.302
≤46.8	17	13		
>46.8	13	17		
**Gender**			0	1
Male	16	16		
Female	14	14		
**Tumor diameter**			14.32	<0.001
Microadenoma	0	5		
Macroadenoma	15	22		
Giant	15	3		
Proliferation*			26.67	<0.001
1/2a	5	25		
1/2b	25	5		
Invasion*			24.86	<0.001
1a/b	7	25		
2a/b	23	5		
Recurrence**			9.93	0.002
Yes	14	3		
No	16	27		

^#^High: According to the median H-score of Jagged1, more than 50%. Low: According to the median H-score of Jagged1, less than 50%. *Five-Tiered prognostic classification of PitNETs: Grade 1a: non-invasive and non-proliferative tumor Grade 1b: non-invasive and proliferative tumor Grade 2a: invasive and non-proliferative tumor Grade 2b: invasive and proliferative tumor Grade 3: metastic tumor (cerebrospinal or systemic metastases) **Definition of recurrence: the occurrence of one or more of the following three conditions: 1. The level of secreted hormones has reached the standard to diagnose PAs again. 2. The MRI imaging has an increase in tumor occupying space. 3. The recurrence of vision and visual field damage.

**Figure 3:**
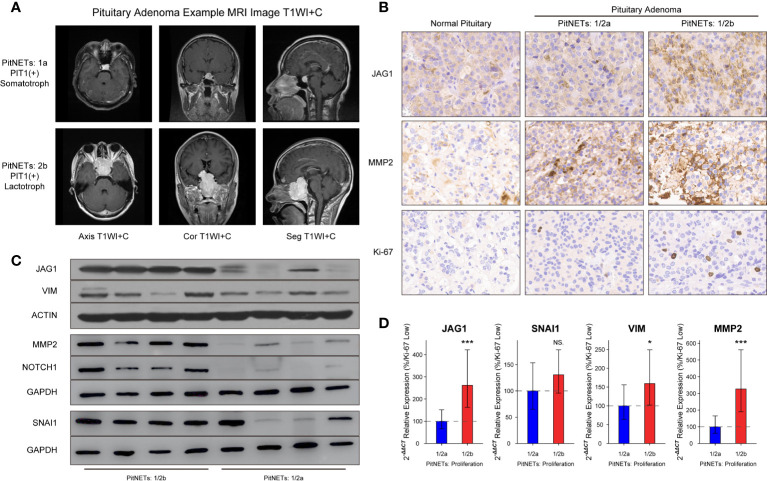
Levels of JAG1 and MMP2 in PA patients. **(A)** MR images of PA patients. **(B)** Bands of western blot on group 1/2a and group 1/2b. **(C)** Immunohistochemistry image of JAG1, MMP2 and Ki-67. **(D)** mRNA levels of JAG1, MMP2, SNAI1, and VIM. *compared to group 1/2a *P*<0.05 ***P<0.001. NS, no sense.

The inclusion criteria of invasion were defined as MR image signs of cavernous or sphenoid sinus invasion in **Figure 3A**. Compared to normal pituitary samples, there are more JAG1-positive cells, MMP2-positive cells, and higher Ki-67 index in PAs in **Figure 3B**. According to the H-score of staining in IHC experiment, there was statistical difference of JAG1 (68.2 ± 7.41 *vs*. 131.7 ± 12.9, *P* = 0.007) and MMP2 levels (105.4 ± 13.7 *vs*. 178.5 ± 26.1, *P* = 0.012) between grade 1/2a and grade 1/2b. The same tendency was verified by western blot experiment and RT-qPCR experiment in **Figures 3C, D**.

### JAG1 Promotes Cell Proliferation, Cell Cycle, and Migration in GH3

**Figure 4:**
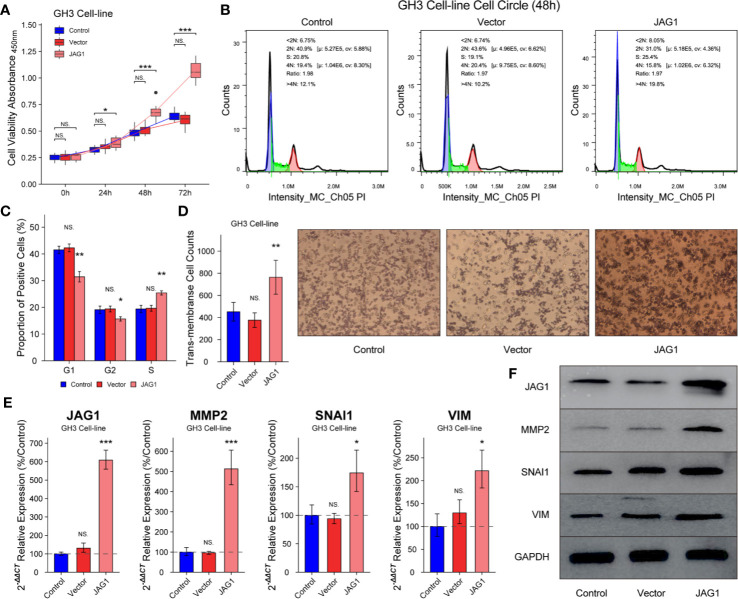
Overexpression JAG1 promoted cell proliferation, cell cycle and migration in GH3 cell line. **(A)** JAG1 promoted the GH3 cell line in the time manner. **(B, C)** JAG1 increased the percent of S phase of GH3 cell line. **(D)** Transwell experiment showed that JAG1 increased the number of trans-membrane positive GH3 cells. **(E)** RT-qPCR experiment showed that JAG1 increased the mRNA levels of MMP2, SNAI1 and VIM in GH3 cells. **(F)** Western blot experiment showed JAG1 increased the protein levels of MMP2, SNAI1 and VIM in GH3 cells. *compared to Vector group *P*<0.05 **P<0.01 ***P<0.001. NS, no sense.

GH3 cells were divided into Control, Vector, and JAG1 group according to transient transfected vector or JAG1 plasmid. The cell proliferation in JAG1 group was 118.3 ± 5.4%(*t* = 1.248, *P* = 0.232), 137.8 ± 6.4% (*t* = 4.57, *P* < 0.001) and 157.3 ± 9.2% (*t* = 10.7, *P* < 0.001) of that in the Vector group after 24, 48, and 72 h transient transfection in **Figure 4A** (*P* < 0.05). Overexpression of JAG1 increased the percent of S phase (25.4 ± 0.8 *vs*. 19.4 ± 1.36%) after 48 h transfection in **Figure 4B** (*P* < 0.01), and statistic data was shown in **Figure 4C** (*P* < 0.01). Trans-membrane positive cells increase to 764 ± 153/field in the JAG1 group from 452 ± 82/field in the Vector group in **Figure 4D** (*P* < 0.001). Western blot and RT-qPCR experiments show the up-regulation of MMP2, SNAI1, and VIM, especially MMP2, after overexpressing the JAG1 for 72 h as shown in **Figures 4E, F** (*P* < 0.05).

### Anti-JAG1 Antibody Inhibits Cell Proliferation, Apoptosis, and Migration in GH3

**Figure 5:**
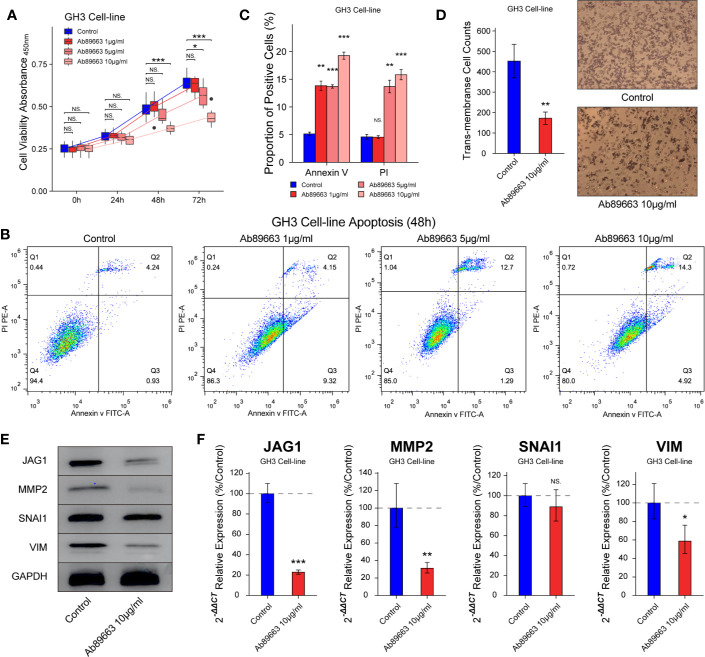
Anti-JAG1 antibody on cell proliferation, apoptosis, and migration of GH3 cell line. **(A)** Anti-JAG1 antibody inhibited the cell viability of GH3 cells in the time and dose manner. **(B, C)** Anti-JAG1 antibody increased the Annexin V positive and PI positive cells in dose manner after 48 h treatment. **(D)** Anti-JAG1 antibody (10 μg/ml) reduced the trans-membrane positive cell after 48 h treatment. **(E)** Western blot experiment showed Anti-JAG1 antibody (10 μg/ml) reduced the protein levels of MMP2, SNAI1, and VIM in GH3 cells. **(F)** RT-qPCR experiment showed that Anti-JAG1 antibody (10 μg/ml) reduced the mRNA levels of MMP2, SNAI1, and VIM in GH3 cells. * compared to control group *P* < 0.05 **P < 0.01 ***P < 0.001. NS, no sense.

In many cancers, small molecules and neutralizing antibodies had been developed for targeting Notch signaling, such as *γ*-secretase inhibitors and antibodies for Notch 1–4. In this study, we verified the effect of anti-JAG1 antibody (Ab89663) on the bioactivity of GH3 cell line. The inhibition of cell proliferation induced by anti-JAG1 antibody was in the manner of time and concentration in **Figure 5A** (*P* < 0.05). Flow cytometry assay showed that anti-JAG1 antibody (10 μg/ml) induced more apoptosis level of GH3 cell than low concentration (1 μg/ml, 5μg/ml) in **Figures 5B, C** (*P* < 0.05). Thence, we chose the concentration (10 μg/ml) for the next experiments. Trans-membrane positive cells was reduced to 172 ± 30/field in 10 μg/ml group from 452 ± 82/field in the control group in **Figure 5D** (*P* < 0.01). Western blot and RT-qPCR experiments show the down-regulation of MMP2, SNAI1, and VIM after anti-JAG1 antibody (10 μg/ml) treatment in **Figures 5E, F** (*P* < 0.05).

### miR-424-3p Is Down-Regulated in PAs and Targeting JAG1 *In Vitro*

**Figure 6:**
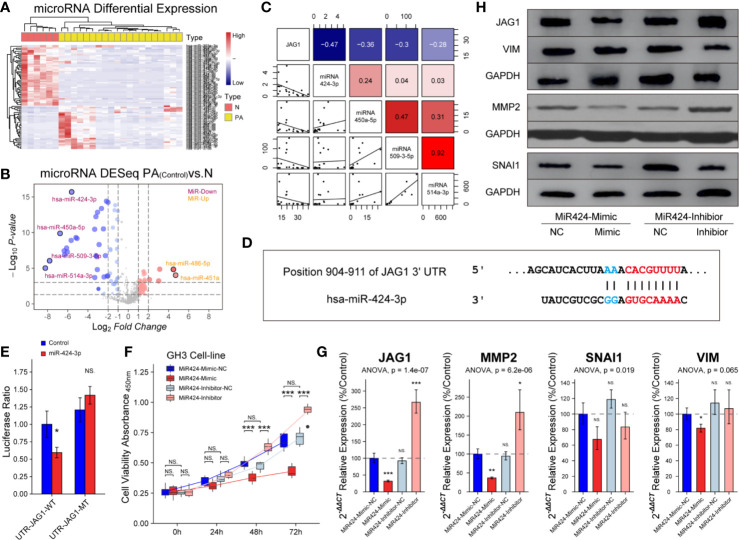
Inhibition of miR-424-3p on GH3 cells by targeting JAG1. **(A)** miRNA differential expression targeting JAG1 between pituitary (n = 6) and patients (n = 20). **(B)** Volcano map of miRNAs showed four down-regulated miRNAs and two up-regulated miRNAs in patients compared to those in normal pituitary. *Log_2_FC* <−2 or >2, *P* < 0.01 **(C)** Correlation analysis between JAG1 and down-regulated miRNAs (miR-424-3p r = −0.47, miR-450a-5p r = −036, miR-509-3-5p, r = −0.3, miR-514a-3p, r = −0.28). *Log_2_FC* <−2 or >2, *P* < 0.01 **(D)** Pattern diagram showed the binding site of JAG1 and miR-424-3p. **(E)** The luciferase assay showed WT-JAG1/MT-JAG1 and miR-424-3p co-transfection in GH3 cells. *compared to control group *P* < 0.05 **(F)** The effect of mimic and inhibitor of miR-424-3p on f GH3 cells. ***compared to NC group *P* < 0.001 **(G)** RT-qPCR experiment showed that mimic and inhibitor of miR-424-3p regulated the mRNA levels of MMP2 and VIM by targeting JAG1 in GH3 cells. *compared to NC group *P* < 0.05 **P < 0.01 ***P < 0.001 **(H)** Western blot experiment showed that mimic and inhibitor of miR-424-3p regulated the protein levels of MMP2 and VIM by targeting JAG1 in GH3 cells. NS, no sense.

miRNAs exert functions through their specific targets and the downstream pathways mediated by the target genes. We investigated which miRNAs were involved in the regulation of JAG1. An unsupervised hierarchical clustering of the miRNAs related to JAG1 revealed two distinct miRNA-expression profiles corresponding to pituitary and PAs in **Figure 6A**. There were four down-regulated miRNAs (miR-424-3p, miR-450a-5p, miR-509-3-5p, and miR-514a-3p) and two up-regulated miRNAs (miR-486-5p and miR-451a) in PAs compared to those in pituitary samples according to Volcano map in **Figure 6B**. The miR-424-3p was filtered for the next functional experiment according to the correlation analysis result in **Figure 6C** (r = −0.47). The luciferase assay showed that the activity of firefly/Renilla luciferase was 0.59 ± 0.08-fold of miR-424-3p-NC group after miR-424-3p and WT-JAG1 co-transfection, but not significantly different after miR-424-3p and MT-JAG1 co-transfection in **Figures 6D, E**. The cell proliferation of the mimic group (30 nmol/L) was reduced to 87.4 ± 5.6, 75.7 ± 4.3, and 63.7 ± 3.9% compared to the corresponding miR-424-3p-NC group (*P* < 0.05), and inhibitor group (100 nmol/L) increased to 107.2 ± 8.4, 130.7 ± 9.4, and 145.4 ± 17.8% compared to the corresponding inhibitor-NC group in **Figure 6F** (*P* < 0.05). Western blot and RT-qPCR experiments showed miR-424-3p inhibited the level of MMP2 and VIM through targeting JAG1 in GH3 cell line in **Figures 6G, H** and **Figure S6** (*P* < 0.05).

## Discussion

As a highly conserved signaling pathway, Notch signaling pathway plays a key role in angiogenesis, cell proliferation, differentiation, and tumor metastasis (20). Previous publications show the differential components of Notch signaling pathway involvement depending on the histopathological type of PAs. However, the characteristic of Notch system functioning in invasion and proliferation of Pas was still unclear. In this study, we found the difference and the role of JAG1 in Notch signaling pathway among three lineage adenomas based on transcription data. According to *in vitro* experiments, JAG1 may promote cell proliferation, migration, and G1/S transition *via* regulating MMP2 and VIM in GH3 cell line.

In various types of cancers, EMT has been associated with the generation of invasive cells and property acquisition of tumor stem cells. EMT-associated gene reprogramming involves key transcription factors in driving this trans-differentiation process (21). PAs containing side population cells that are depleted from endothelial and immune cells, express the properties of tumor stem cell, including EMT-linked factors (22). Gonadotroph adenomas of different growth rates present different gene expression profiles: the higher expression levels of genes are related to EMT resting in the fast-growing tumors (23). Our research is the first study to investigate genome-wide mRNA expression based on Five-Tiered prognostic classification of PitNETs. Using the criteria of Five-Tiered prognostic classification, we revealed two distinct gene expression profiles and concluded that EMT was the major factor of tumor proliferation and migration in PAs, especially in PIT1 lineage adenomas, followed by corticotroph adenoma.

Through the diversity of TPM, the top three pathways were extracellular matrix–receptor interaction, Notch signaling pathway, and protein digestion and absorption by KEGG pathway enrichment. The authors chose to focus mostly on Notch signal pathway regard to markers of tumor proliferation and invasion. Volcano map and GSEA both demonstrated the priority of Notch signaling pathway in PitNETs 1/2b: Notch2 and JAG1 in PIT1 linage; Notch3 and JAG1 in corticotroph; Notch3 and JAG1 in gonadotroph. We speculated that the downstream key ligand was JAG1, instead of DLLs, in three lineage adenomas, although upstream Notch receptor was different according to histopathological type of PAs. Furthermore, we extracted the mRNA quantification of intact Notch signal pathway, filtered DLL1, DLL4, DTX4, HES1/2/5, JAG1, and HEYL with statistical difference. PFS analysis results of DLL1 (Hazard Ratio = 3.82, 95% CI: 1.47–9.91, P = 0.011) and HES5 (Hazard Ratio = 4.07, 95% CI: 1.56–10.6, P = 0.007) were also interesting, but the result of JAG1 (Hazard ratio =4.59, 95%CI:1.77–11.87, P = 0.006) seemed more significant. Univariate analysis of JAG1 expression and clinico-pathological characteristics suggested that JAG1 facilitates the invasion and proliferation of PAs.

As a key ligand of Notch signaling pathway, high level of JAG1 was found in breast cancer, ovarian cancer and metastatic prostate cancer and was linked to poor survival rate (24). The level of JAG1 positively controlled the activation of hepatic stellate cells (HSCs), and migration of HSCS could be noticeably restrained by knockdown of JAG1. Down-regulation and up-regulation of JAG1 inhibited and promoted, respectively, HSC activation. The migratory capacity of HSCs was markedly restrained by JAG1 siRNA (25). These reports coincide with our finding that JAG1 mediated adenomas progression by modulating cell proliferation and migration. Overexpression of JAG1 greatly increased the levels of MMP2 and VIM *in vitro* experiment. Furthermore, the number of trans-membrane positive cells indicates that JAG1 promoted the migration and invasion of GH3 cells.

Inhibiting Notch signaling pathway becomes a promising anti-cancer strategy for its critical role in cancer stem cell maintenance and tumor angiogenesis, usually including Notch inhibitor and antibodies (26). A phase Ib trial of Demcizumab targeted DLL4 combined with standard chemotherapy resulted in 50% patients with objective tumor response by regulating Notch signaling and angiogenesis in metastatic non-squamous non-small cell lung cancer (27). Enoticumab, binding human Dll4 and disrupting Notch-mediated signaling, had antitumor activity in molecular- and angiogenesis-relevant scenarios in ovarian cancer and other solid tumors (28). In this study, we noticed the inhibition of antibody-JAG1 in GH3 cell line and further traced the upstream epigenetic proof to be potent treatment.

MicroRNAs (miRNAs) regulated the function of eukaryotic messenger RNAs through pairing complementary sequences in the mRNA’s 3′-untranslated region (3′UTR) (29). In theory, miRNAs are ideal therapeutic choice for cancers because their small size enables them to penetrate the dense architecture of cancer (30). We reported the circulating miRNAs profile of 169 miRNAs differently expressed between somatotroph adenomas and healthy samples (31). *In vitro* experiments verified that miR-424-3p relieved the migration and invasion of GH3 cell line, mimics or inhibitor of miR-424-3p can regulate the level of JAG1 which, in turn, affects cell proliferation and the levels of MMP2 and VIM in GH3 cell line, respectively.

In conclusion, our research investigates the role of Notch signal pathway in cell proliferation and EMT in PAs. It also provides the potential PA treatment through targeting JAG1. Medical treatment could be a good choice for patients who decline surgery or unlikely attain biochemical cure, although the guidelines indicate that transsphenoidal surgery is the preferred treatment for patients with primary PA. Combining miRNA-424-3p loaded nanoparticle or anti-JAG1 antibody could be an effective method for treatment strategy to PitNETs 1/2b adenomas, especially for patients with over-activation of Notch signaling and resistant to standard treatment.

## Data Availability Statement

The original contributions presented in the study are deposited in the Baidu Netdisk. This data can be found here: https://pan.baidu.com/s/1-afZGEcQx6EExMD3uKtDgg, extraction code: JAG1.

## Ethics Statement

The studies involving human participants were reviewed and approved by the Internal Review Board of Beijing Tiantan Hospital, which was affiliated to Capital Medical University and conformed to the ethical guidelines of the Declaration of Helsinki (no. KY-2013-02). The patients/participants provided their written informed consent to participate in this study.

## Author Contributions

YC was responsible for design and writing. BL and QF performed histological examination. JF analyzed and interpreted the transcriptome data. JC and WX performed RT-qPCR and western blot experiment. CL and SC analyzed and interpreted the follow-up data. YZ revised the manuscript. HG designed the protocol and revised the manuscript. All authors contributed to the article and approved the submitted version.

## Funding

This work was financially supported by the Beijing Natural Science Foundation of China (7162035), the Beijing High-level Talent Plan (2015–3–040), the National Natural Science Foundation of China (81602182, 81702455).

## Conflict of Interest

The authors declare that the research was conducted in the absence of any commercial or financial relationships that could be construed as a potential conflict of interest.

## Supplementary Material

Supplementary Figure 1 Heatmap of unsupervised hierarchical clustering based on different lineage adenomas. **(A)** Corticotroph adenomas. **(B)** Gonadotroph adenomas. **(C)** Pit-1 lineage adenomas.

Supplementary Figure 2 Volcano map of transcriptome and GSEA multiple pathways of Notch pathway. **(A)** Volcano map of transcriptome in corticotroph adenomas. **(B)** Volcano map of transcriptome in gonadotroph adenomas. **(C)** Volcano map of transcriptome in Pit-1 lineage adenomas. **(D)** GSEA multiple pathways analysis of Notch pathway in corticotroph adenomas. **(E)** GSEA multiple pathways analysis of Notch pathway in gonadotroph adenomas. **(F)** GSEA multiple pathways analysis of Notch pathway in Pit-1 lineage adenomas.

Supplementary Figure 3 mRNA levels of Notch signal pathway in 60 patients. 16 up-regulated genes of Notch signal pathway, including all Notch receptors.

Supplementary Figure 4 Correlation analysis of Notch signal pathway with genes related to EMT in 60 patients.

Supplementary Figure 5 PFS time according to Notch signal pathway in 60 patients. 6 Notch signal pathway genes which shorted the PFS time in PA patients: DLL1, HES5, JAG1, KAT2A, Notch1 and Notch4.

Supplementary Figure 6 RT-qPCR experiment showed that mimic and inhibitor of miR-424-3p on regulated the mRNA levels of MMP2 and VIM by targeting JAG1 in GH3 cells. *compared to NC group *P* < 0.05 ***P* < 0.01 ****P* < 0.001.
