# Supplementary figures and images for: JAG1, Regulated by microRNA-424-3p, Involved in Tumorigenesis and Epithelial–Mesenchymal Transition of High Proliferative Potential-Pituitary Adenomas
Source: Front Oncol. 2020 Dec 23;10:567021. doi: 10.3389/fonc.2020.567021 (PMC7787033; doi:10.3389/fonc.2020.567021)

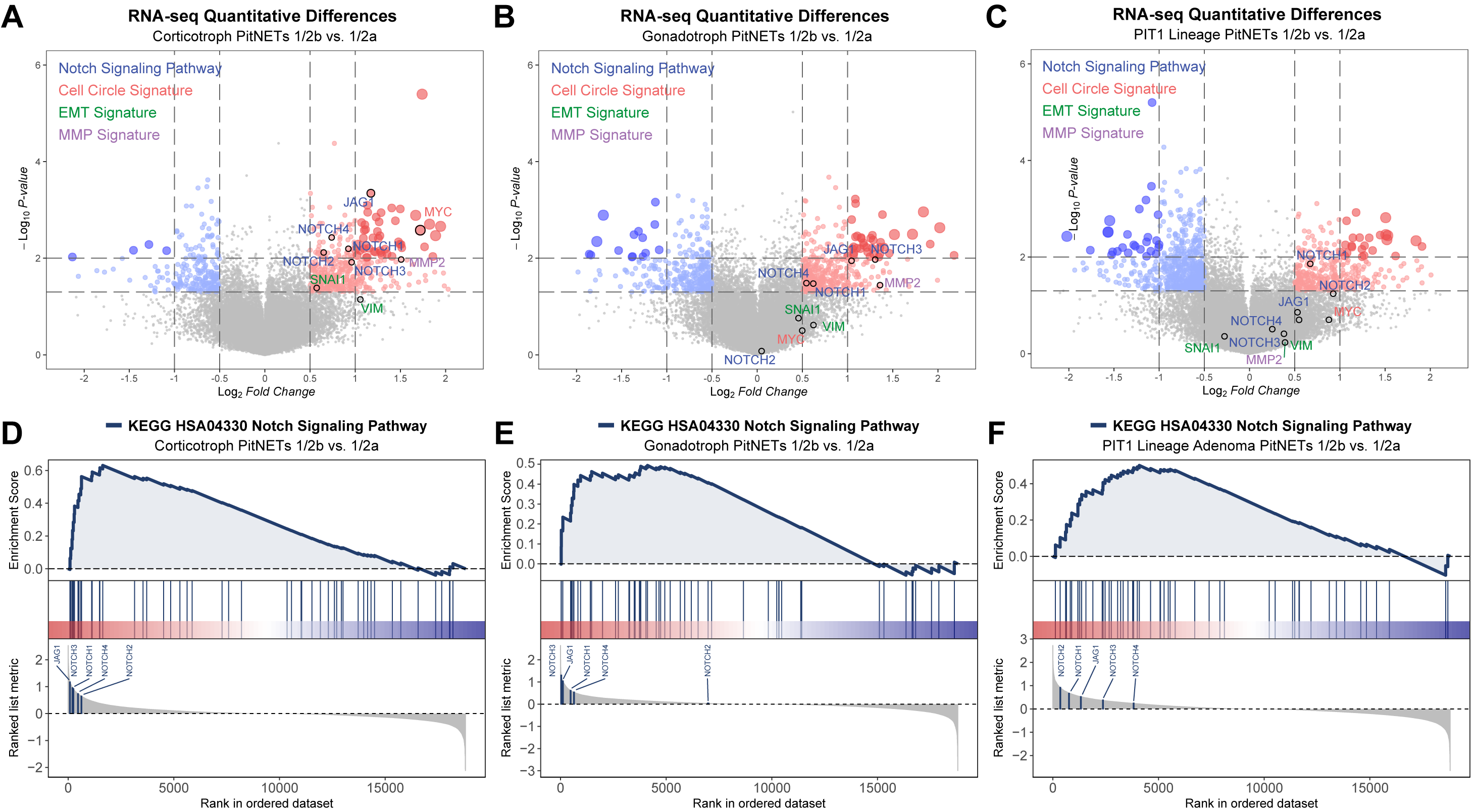

Supplement: Supplementary Figure 1 — Heatmap of unsupervised hierarchical clustering based on different lineage adenomas. (A) Corticotroph adenomas. (B) Gonadotroph adenomas. (C) Pit-1 lineage adenomas. [file Image_1.tif]

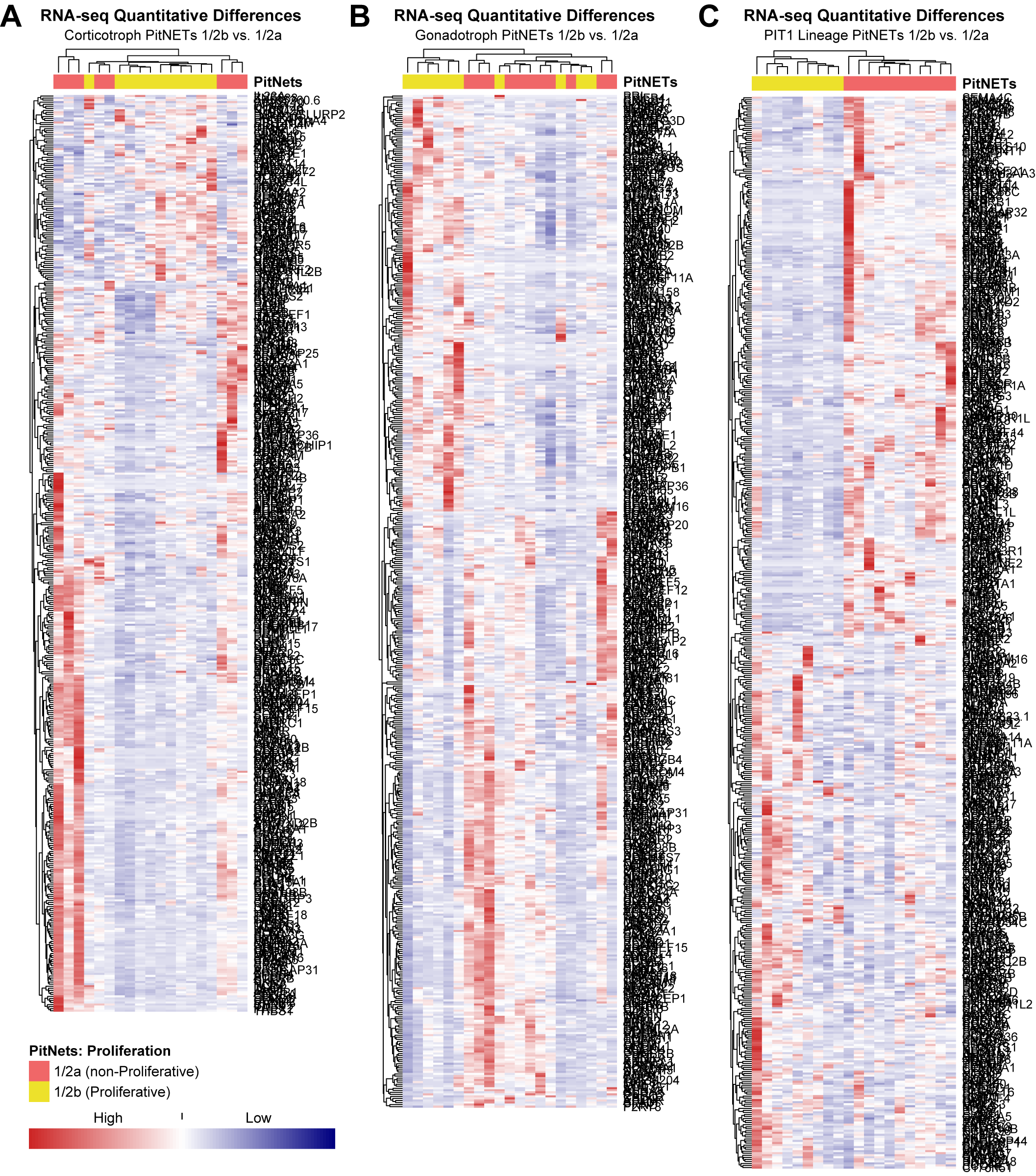

Supplement: Supplementary Figure 2 — Volcano map of transcriptome and GSEA multiple pathways of Notch pathway. (A) Volcano map of transcriptome in corticotroph adenomas. (B) Volcano map of transcriptome in gonadotroph adenomas. (C) Volcano map of transcriptome in Pit-1 lineage adenomas. (D) GSEA multiple pathways analysis of Notch pathway in corticotroph adenomas. (E) GSEA multiple pathways analysis of Notch pathway in gonadotroph adenomas. (F) GSEA multiple pathways analysis of Notch pathway in Pit-1 lineage adenomas. [file Image_2.tif]

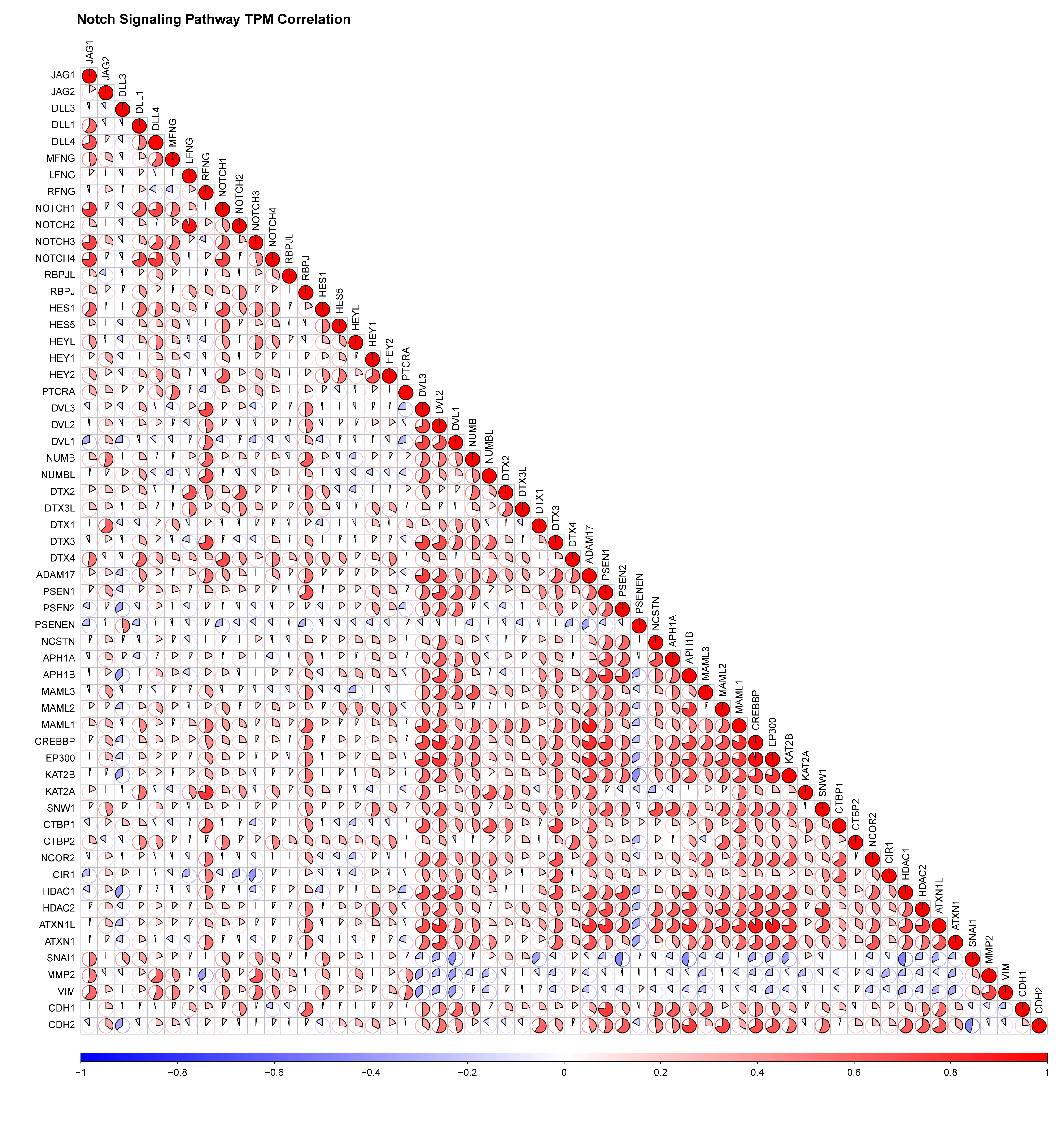

Supplement: Supplementary Figure 3 — mRNA levels of Notch signal pathway in 60 patients. 16 up-regulated genes of Notch signal pathway, including all Notch receptors. [file Image_3.tif]

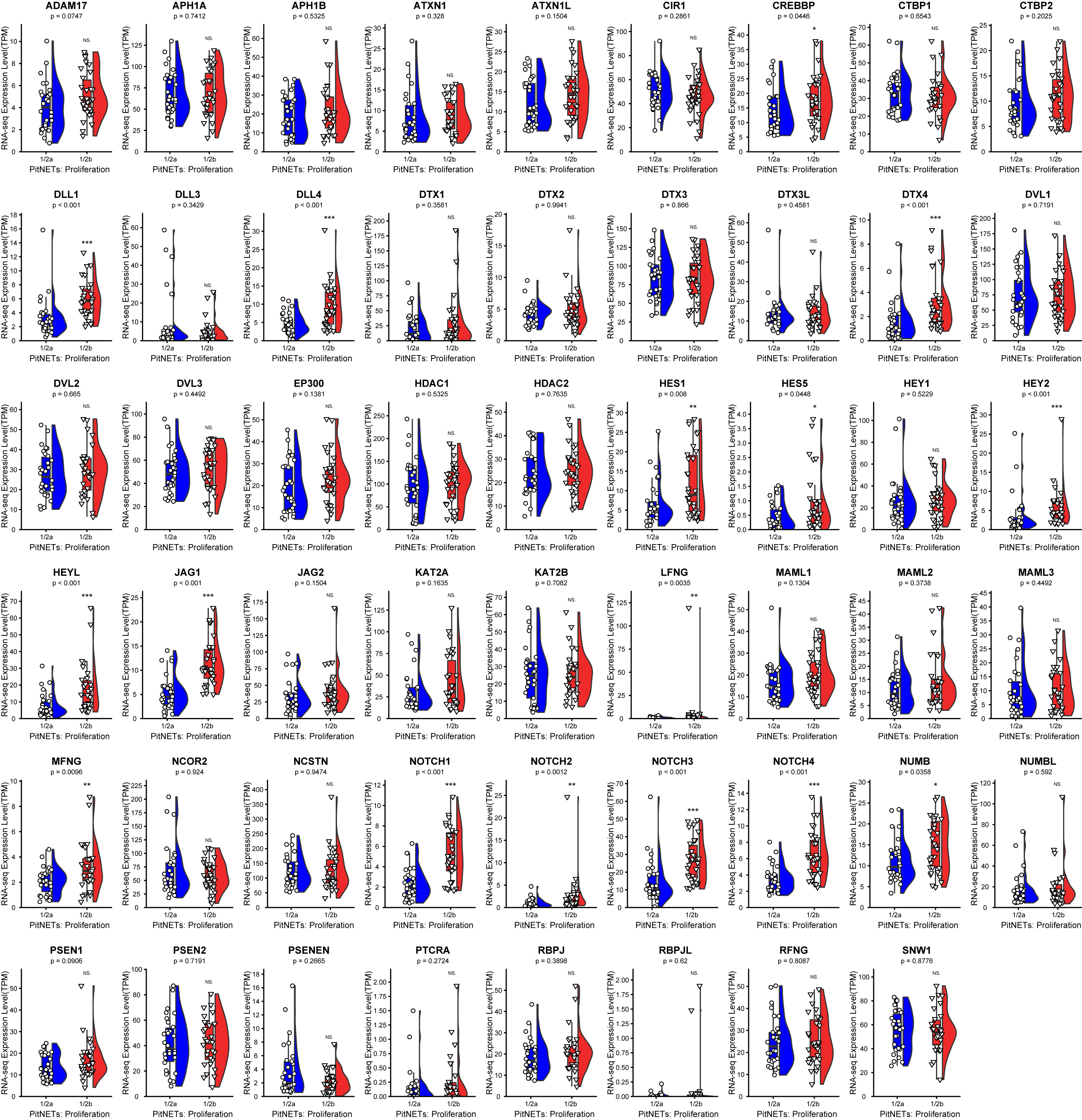

Supplement: Supplementary Figure 4 — Correlation analysis of Notch signal pathway with genes related to EMT in 60 patients. [file Image_4.tif]

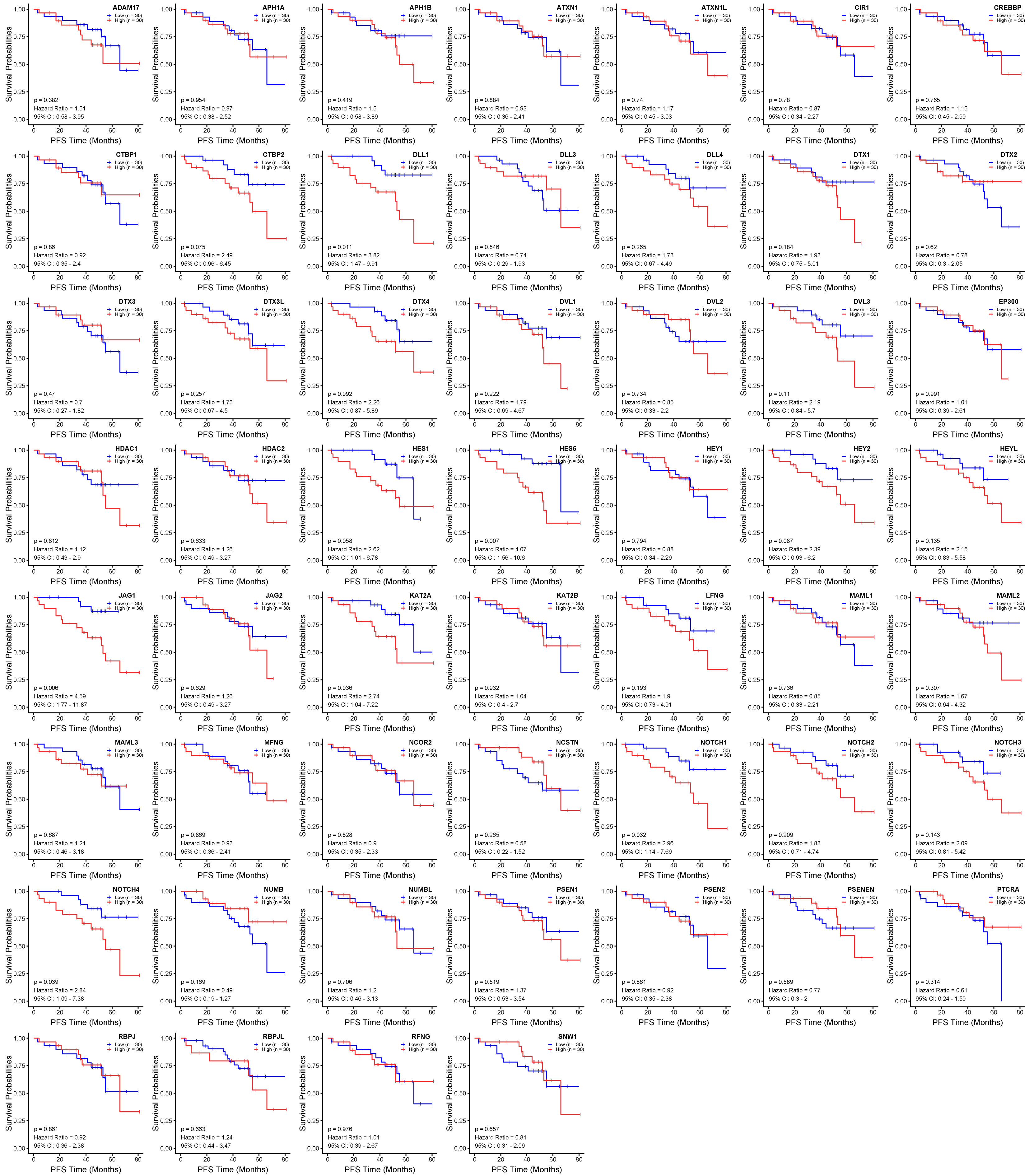

Supplement: Supplementary Figure 5 — PFS time according to Notch signal pathway in 60 patients. 6 Notch signal pathway genes which shorted the PFS time in PA patients: DLL1, HES5, JAG1, KAT2A, Notch1 and Notch4. [file Image_5.tif]

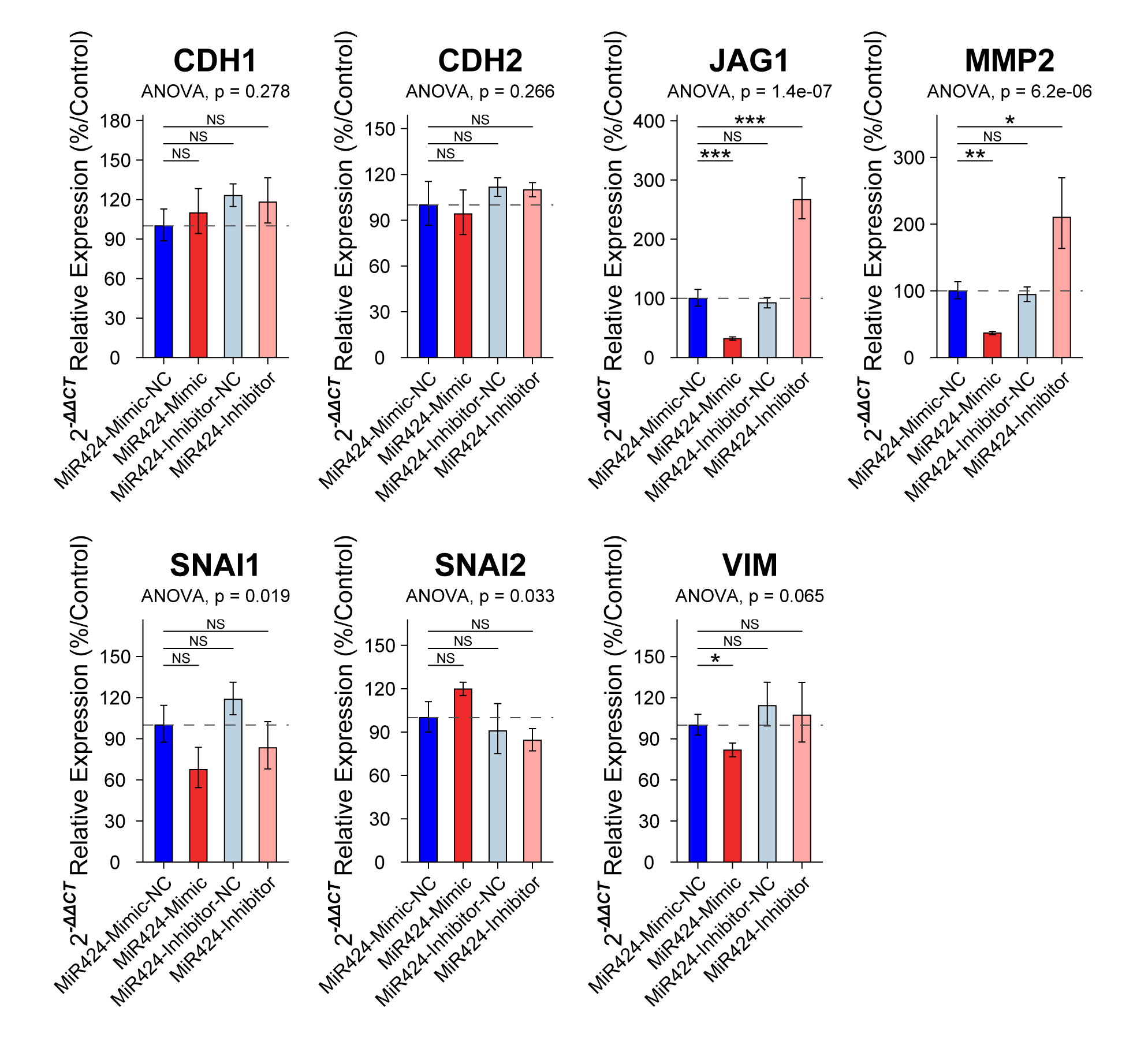

Supplement: Supplementary Figure 6 — RT-qPCR experiment showed that mimic and inhibitor of miR-424-3p on regulated the mRNA levels of MMP2 and VIM by targeting JAG1 in GH3 cells. *compared to NC group P < 0.05 **P < 0.01 ***P < 0.001. [file Image_6.tif]
