# Supplementary material for: JAG1, Regulated by microRNA-424-3p, Involved in Tumorigenesis and Epithelial–Mesenchymal Transition of High Proliferative Potential-Pituitary Adenomas
Source: Front Oncol. 2020 Dec 23;10:567021. doi: 10.3389/fonc.2020.567021 (PMC7787033; doi:10.3389/fonc.2020.567021)
Supplement: Supplementary file 7 [file Table_1.docx]

**Table1** Primers in RT-PCR experiment

| **Gene** | **Up:** | **Down:** |
| --- | --- | --- |
| hJAG1 | gggtcagttcgagttggaga | gcgggactgatactccttga |
| rJAG1 | aggggccgatttgtactcaa | caaaggcacaaggggaagac |
| hMMP 2 | cagccctgcaagtttccatt | gttgcccaggaaagtgaagg |
| rMMP 2 | catcaaatcggactggctgg | caggtgaaggagaaggctga |
| hSnail | agtggttcttctgcgctact | gtagggctgctggaaggtaa |
| rSnail | agagtgcctttgtaccctcc | ggagggatgggactattgca |
| hVIM | gagtccactgagtaccggag | acgagccatttcctccttca |
| rVIM | tgacattgagatcgccacct | tcatcgtggtgctgagaagt |
| hGAPDH | cacatcgctcagacaccatg | tgacggtgccatggaatttg |
| rGAPDH | gagacagccgcatcttcttg | tgactgtgccgttgaacttg |
